# Supplementary material for: Preparation of Affinity Purified Antibodies against ε-Glutaryl-Lysine Residues in Proteins for Investigation of Glutarylated Proteins in Animal Tissues
Source: Biomolecules. 2021 Aug 7;11(8):1168. doi: 10.3390/biom11081168 (PMC8394851; doi:10.3390/biom11081168)
Supplement: Supplementary file 1 [file biomolecules-11-01168-s001.zip › biomolecules-1263476-supplementary.pdf]

Preparation of Affinity Purified Antibodies Against ε-Glutaryllysine Residues in Proteins for Investigation of Glutarylated Proteins in Animal Tissues

Ekaterina F. Kolesanova[1]*, Aleksandra I. Boyko[2], Anastasiya A. Chashnikova[2], Sergei .N. Gnedoy[3], Thilo Kaehne[4], Daria A. Ivanova[1], Alyona V. Kolesnichenko[1], Vasily A. Aleshin[2,5], Artem V. Artiukhov[2,5], Victoria I. Bunik[2,5,6]*

[1] Institute of Biomedical Chemistry, Moscow, Russia; ekaterina.kolesanova@ibmc.msk.ru
[2] Faculty of Bioengineering and Bioinformatics, Lomonosov Moscow State University, 119991 Moscow, Russia; bunik@belozersky.msu.ru
[3] "Agrobiomed" Ltd., 249010, Borovsk, Kaluga region, Russia; sngnedoy@mail.ru
[4] Institute of Experimental Internal Medicine, Otto-von-Guericke University, 39120 Magdeburg, Germany
[5] Belozersky Institute of Physico-Chemical Biology, Lomonosov Moscow State University 119991 Moscow, Russia
[6] Department of Biological Chemistry, Sechenov First Moscow State Medical University, 119146 Moscow, Russia
* Correspondence: E.F.K.,ekaterina.kolesanova@ibmc.msk.ru; Tel.: +7 499 246 3375; V.I.B. bunik@belozersky.msu.ru

A

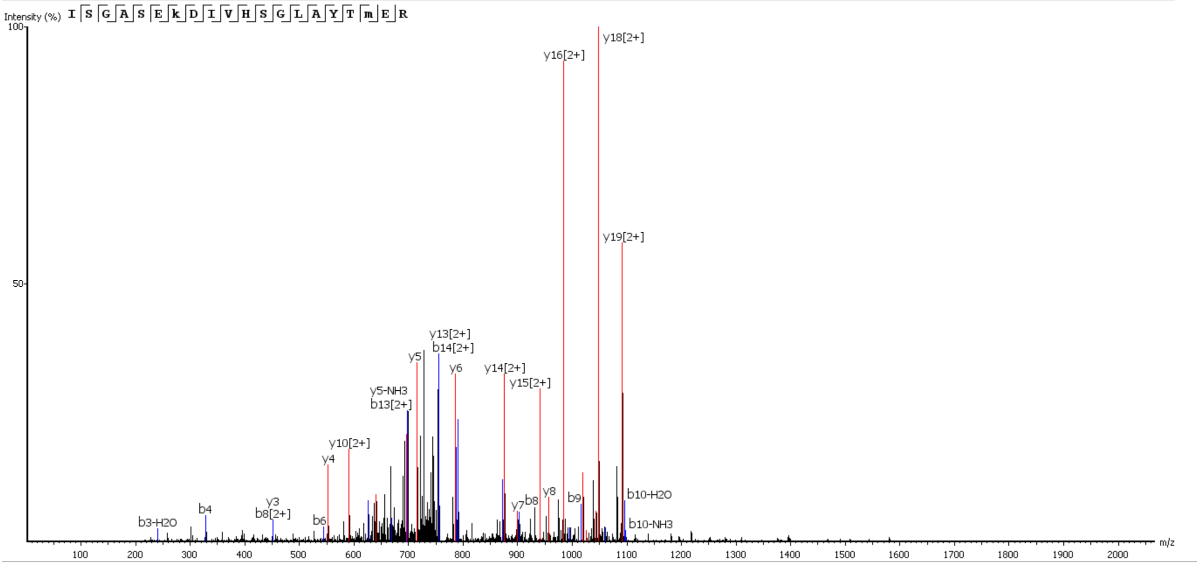

B

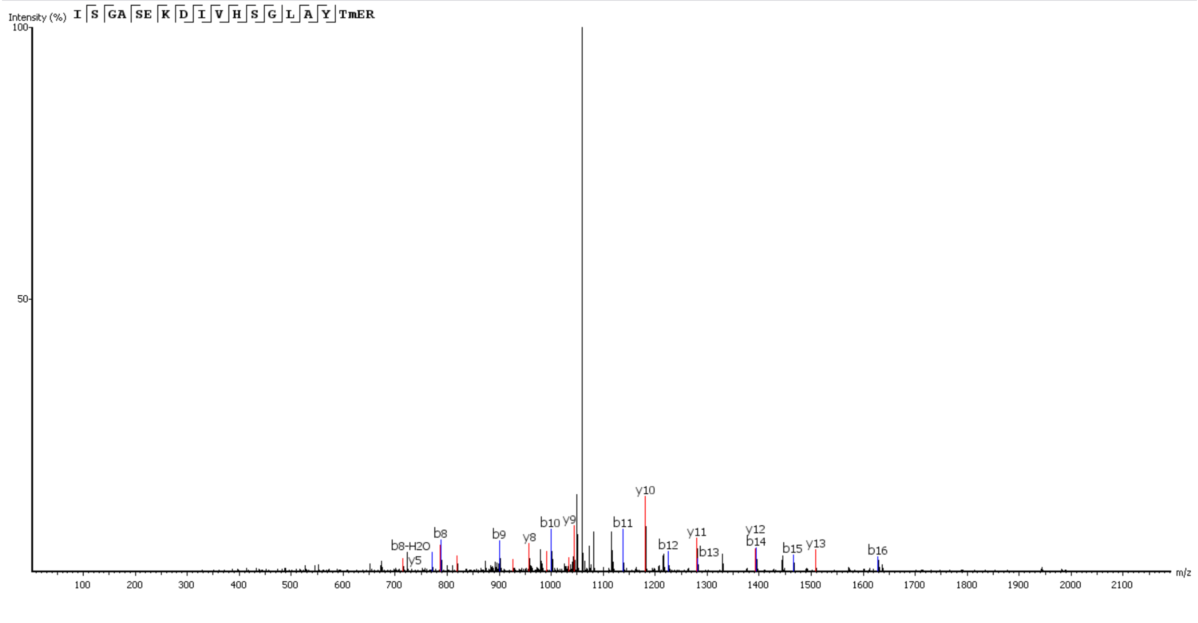

Figure S1. Representative MS/MS spectra of the GDH peptides containing K503 residue. (**A**) – the peptide with glutarylated K503; (**B**) – the peptide comprising non-glutarylated K503. The images of the spectra were prepared in PEAKS Studio 8.0. Glutarylation is confirmed by the increase of 114.03 (for

single-charged ions) or 57.02 (for double-charged ions) in fragment ion monoisotopic m/e values compared to fragment ion m/e values in MS/MS spectrum of the non-glutarylated peptide.
